# The Role of Na^+^ and K^+^ Transporters in Salt Stress Adaptation in Glycophytes

**DOI:** 10.3389/fphys.2017.00509

**Published:** 2017-07-18

**Authors:** Dekoum V. M. Assaha, Akihiro Ueda, Hirofumi Saneoka, Rashid Al-Yahyai, Mahmoud W. Yaish

**Affiliations:** ^1^Department of Biology, College of Science, Sultan Qaboos University Muscat, Oman; ^2^Graduate School of Biosphere Science, Hiroshima University Hiroshima, Japan; ^3^Department of Crop Sciences, College of Agricultural and Marine Sciences, Sultan Qaboos University Muscat, Oman

**Keywords:** salinity, abiotic stress, sodium transporters, potassium transporters, glycophytes, halophytes

## Abstract

Ionic stress is one of the most important components of salinity and is brought about by excess Na^+^ accumulation, especially in the aerial parts of plants. Since Na^+^ interferes with K^+^ homeostasis, and especially given its involvement in numerous metabolic processes, maintaining a balanced cytosolic Na^+^/K^+^ ratio has become a key salinity tolerance mechanism. Achieving this homeostatic balance requires the activity of Na^+^ and K^+^ transporters and/or channels. The mechanism of Na^+^ and K^+^ uptake and translocation in glycophytes and halophytes is essentially the same, but glycophytes are more susceptible to ionic stress than halophytes. The transport mechanisms involve Na^+^ and/or K^+^ transporters and channels as well as non-selective cation channels. Thus, the question arises of whether the difference in salt tolerance between glycophytes and halophytes could be the result of differences in the proteins or in the expression of genes coding the transporters. The aim of this review is to seek answers to this question by examining the role of major Na^+^ and K^+^ transporters and channels in Na^+^ and K^+^ uptake, translocation and intracellular homeostasis in glycophytes. It turns out that these transporters and channels are equally important for the adaptation of glycophytes as they are for halophytes, but differential gene expression, structural differences in the proteins (single nucleotide substitutions, impacting affinity) and post-translational modifications (phosphorylation) account for the differences in their activity and hence the differences in tolerance between the two groups. Furthermore, lack of the ability to maintain stable plasma membrane (PM) potentials following Na^+^-induced depolarization is also crucial for salt stress tolerance. This stable membrane potential is sustained by the activity of Na^+^/H^+^ antiporters such as SOS1 at the PM. Moreover, novel regulators of Na^+^ and K^+^ transport pathways including the Nax1 and Nax2 loci regulation of SOS1 expression and activity in the stele, and haem oxygenase involvement in stabilizing membrane potential by activating H^+^-ATPase activity, favorable for K^+^ uptake through HAK/AKT1, have been shown and are discussed.

## Introduction

High salinity, a global threat to agricultural production, is a multicomponent stress under the control of multitudes of genes and gene networks. The first component of this stress is osmotic, and arises from NaCl-induced reduction of the solute potential of the soil solution, which in turn reduces the hydraulic conductance and hence water and solute uptake by plants (Munns and Tester, 2008). The second component is ionic (ion toxicity), which arises from accumulation of noxious quantities of Na^+^ in the cells and tissues of the plant that adversely affect its growth and development. Unlike in animals, Na^+^ is a non-essential element in plants (except in some C_4_ plants) (Kronzucker et al., 2013; Nieves-Cordones et al., 2016a), and its excess accumulation will be highly deleterious to the plants, with effects including induction of cytosolic K^+^ efflux and consequently an imbalance in cellular homeostasis, oxidative stress, interference with Ca^2+^ and K^+^ functions, nutrient deficiency, retarded growth and even the death of plant cells (Tester and Davenport, 2003; Munns and Tester, 2008; Craig Plett and Møller, 2010; Cabot et al., 2014).

It is a combination of these effects that leads to a decline in crop production worldwide, since most croplands are becoming increasingly saline due to poor irrigation and climate change-related events. Since most crops are glycophytes (plants that are adapted to low-Na^+^ environments), the surge in research to unravel plants' adaptive responses to salt stress, especially stress-responsive genes that can be explored to generate stress-tolerant crops, is not surprising, and the possibility of exploring halophytic genes to enhance salt stress tolerance in glycophytes is equally appealing, although not without limitations. These limitations include, for example, differences in post-translational regulation between H^+^-ATPases in halophytes and glycophytes, and the lack of Na^+^ sequestration ability of *Aeluropus littoralis* AlNHX in pea plants (Himabindu et al., 2016). The main contributing factor to salt stress is Na^+^, and the genes encoding Na^+^ transporters and channels have been the major targets for engineering salt stress tolerance. For salt-accumulating halophytes that actively accumulate Na^+^ in the leaves and use it as an osmoticum, the down-regulation of Na^+^ uptake transporters will be deleterious (Flowers and Colmer, 2008; Katschnig et al., 2015). However, for Na^+^-excluding halophytes and glycophytes, this would to a large extent be a favorable trait. This is especially important for glycophytes that are very susceptible to salt stress and can hardly tolerate salt stress greater than 100 mM NaCl stress. Most crops fall into this category of plants (Yokoi et al., 2002), and thus understanding how they cope in Na^+^-rich environments is important for engineering salinity tolerance. Cation transporters and channels have been shown to be involved in Na^+^ and K^+^ homeostasis in plants. While some of these transporters mediate the accumulation of tolerable quantities of Na^+^ in the plants, others mediate a noxious quantity that seriously affects the growth of the plants (Horie et al., 2001, 2007; Suzuki et al., 2016b).

The regulation of Na^+^ uptake and transport in plants under salt stress has been widely interpreted in the context of maintaining high tissue K^+^/Na^+^ ratios, and hence high cytosolic K^+^/Na^+^ ratios, which has become a key salt tolerance trait (Shabala and Pottosin, 2014). This interpretation is logical because K^+^ participates in a myriad of physiological functions in plants, and since high external Na^+^ often competitively inhibits its uptake, K^+^ deficiency becomes severe under salt stress, leading to growth impairment. This means that salt stress sensitivity is almost exclusively attributable to K^+^ deficiency, especially given that K^+^ concentration in soil is usually in the micromolar range (Véry and Sentenac, 2003). Thus, much research has focused recently on unraveling the mechanisms of plant adaptation to low-K^+^ conditions in the presence or absence of salt stress, which can be explored to enhance salt stress tolerance. It follows from these studies that high K^+^ availability ameliorates plant growth under salt stress, but under low K^+^ salt stress is devastating.

One significant effect of salt stress on K^+^ homeostasis is Na^+^-induced K^+^ efflux from both root and leaf cells (Wang et al., 2009; Demidchik et al., 2014). This efflux has been clearly established to be the result of excess Na^+^ influx into the cytoplasm, leading to depolarization of the membrane potential below resting potential, with a consequential activation of K^+^ outward rectifier channels, such as GORK (guard cell outward rectifying K^+^ channel), through which K^+^ is extruded. Thus, the ability of plant cells to prevent membrane depolarization by maintaining a more negative inside potential, in order to enhance intracellular K^+^ retention (inhibition of K^+^ efflux) (Falhof et al., 2016), will constitute a stress tolerance mechanism. The capacity to retain intracellular K^+^ has been recently put forward as equally crucial for salt stress tolerance (Janicka-Russak and Kabała, 2015). An important question to ask here is: how is negative inside membrane potential maintained under salt stress to achieve this positive trait? Recent studies show that PM H^+^-ATPases and tonoplast (TP) H^+^-ATPases/H^+^-PPases, K^+^ transporters, Na^+^/H^+^ exchangers (NHX) and the antiporter, salt overly sensitive 1 (SOS1) are important players in the process, acting in concert to abate salt stress and low-K^+^ effects on plant growth (Pottosin and Dobrovinskaya, 2014; Janicka-Russak and Kabała, 2015; Falhof et al., 2016). Thus, if crops are imbued with these mechanisms, then survival and sustained production under these adverse conditions will be assured.

It thus becomes clear that regulating toxic Na^+^ accumulation and enhancement of K^+^ uptake and accumulation are keys to the survival of glycophytes in saline environments, implying that Na^+^ and K^+^ transporters and channels should play a major role in the salt stress tolerance of these plants. Transcriptomics is a useful tool in mining novel putative pathways underlying salt stress tolerance in plants, which can be studied in detail to understand the adaptive mechanisms. Recently we (Yaish et al., 2017) studied differentially-expressed genes in the root and leaf of date palm (*Phoenix dactylifera* L.) under salt stress, using RNA-sequencing, and found that Na^+^ and K^+^ transporters were differentially expressed, especially in the root, indicating that they could be involved in salt adaptation of the plant. We are presently characterizing the functions of these transporters to test this hypothesis. This review seeks to document the role of major cation transporters and channels in salt stress tolerance in glycophytes, which will help answer one fundamental question: what accounts for differential salt tolerance between salt-tolerant and salt-sensitive glycophytes, or between glycophytes and halophytes in the context of Na^+^ and K^+^ homeostasis? To answer this question, we will examine the participation of Na^+^ and K^+^ transporters in regulating Na^+^ and K^+^ uptake and translocation to achieve an optimal Na^+^/K^+^ ratio, which is a key to salt stress tolerance. Worthy of interest also will be to assess how they intervene in other intracellular processes, such as pH regulation, and vesicle and protein trafficking. Research on Na^+^ and K^+^ homeostasis in plants has significantly increased our understanding of salt stress tolerance mechanisms, but at the same time revealed the complexity of salt stress tolerance by uncovering new downstream regulators of Na^+^ and K^+^ uptake and transport components.

## Sodium homeostasis

Uptake of Na^+^ at the root-soil boundary is believed to occur mainly through non-selective cation channels (NSCC), including the cyclic nucleotide-gated channels (CNGCs) and glutamate receptors (GLRs), as well as through some high affinity K^+^ transporters (HKTs) and K^+^ channels including the Arabidopsis K^+^ transporter (AKT1), and the high-affinity K^+^ uptake transporter (HAK) (Tester and Davenport, 2003; Craig Plett and Møller, 2010; Hanin et al., 2016). Also, there is mounting evidence that aquaporins are also implicated in Na^+^ uptake in plants (Byrt et al., 2017). The Na^+^ absorbed by the root then moves to the xylem with the aid of other transporters and channels and is delivered to the shoot, especially the leaf blade, where its effects are most felt (Munns and Tester, 2008). In glycophytes, regulating this transport of Na^+^ to the leaf is crucial and will be a determinant in their adaptation to salt stress (Tester and Davenport, 2003). Therefore, transporters that operate to reverse the processes involved in Na^+^ uptake and translocation would be relevant, although Na^+^ uptake, translocation and accumulation in the leaf is not always correlated with salt stress sensitivity. For example barley plants tolerate salt stress by accumulating salt in the leaves for osmotic adjustment (Mian et al., 2011a).

## Mechanisms restricting Na^+^ uptake determine tolerance to salinity

Limitation of Na^+^ uptake at the root may require the down-regulation of genes coding for Na^+^ influx transporters or channels at the soil-root boundary. CNGCs are a class of non-selective cation channels that are permeable to monovalent and divalent cations such as Na^+^, K^+^, and Ca^2+^ (Demidchik and Maathuis, 2007; Mian et al., 2011b; Hanin et al., 2016). Although their down-regulation can prevent Na^+^ uptake, it can potentially be concomitantly harmful to the plants, as uptake of other elements will be compromised. However, in rice root, the down-regulation of the rice (*Oryza sativa*) *OsCNGC1* contributed to the superior tolerance of the cultivar FL478 to salt stress (Senadheera et al., 2009), as it could avert toxic Na^+^ influx, in contrast with the sensitive cultivar, in which the gene was up-regulated by salinity stress. Also, *Arabidopsis thaliana* null mutants, *Atcngc10*, were found to have enhanced growth under salt stress compared to wild-type plants (Jin et al., 2015). Furthermore, Atcngc3 T-DNA insertion mutants showed an increase in tolerance to high levels of NaCl and KCl (Gobert et al., 2006). With regard to the correlation between CNGC down-regulation and stress tolerance, Mekawy et al. (2015) evaluated the relative tolerance of two rice cultivars, Egyptian Yasmine and Sakha 102. They observed that the greater tolerance of Egyptian Yasmine was partially attributable to the down-regulation of *OsCNGC1*, with the concomitant up-regulation of plasma membrane protein 3 (PMP3), a plasma membrane protein involved in the inhibition of excess Na^+^ uptake at the level of the root (Inada et al., 2005). Plant aquaporins are crucial for water homeostasis, transport of solutes such as glycerol, CO_2_, urea, H_2_O_2_, ammonia, boron and silicon, salt stress tolerance (Chaumont and Tyerman, 2014; Chevalier and Chaumont, 2014; Li G. et al., 2014; Mansour, 2014). In animals, some aquaporins also mediate ion transport (Ikeda et al., 2002), which prompted investigation of possible similar roles in plants. It was not until recently that such a role was shown to be indeed present in plants, at least in Arabidopsis (Byrt et al., 2017) where the phenomenon was evaluated. In this study, electrophysiological studies using Xenopus oocytes and yeast complementation tests showed that *Arabidopsis thaliana* plasma membrane intrinsic protein AtPIP2;1 has strong Na^+^ conductance, although it was not clear from the study whether it is Na^+^-selective or not. This finding would suggest that if orthologs of this member are found in all other plants including glycophytes, they would be a potential source of toxic Na^+^ influx in the plants, especially given that in some plants aquaporin genes are induced by water deficit conditions which is also characteristic of salt stress conditions (Chaumont and Tyerman, 2014). However, overexpression of the banana (*Musa acuminata*) *MaPIP1;1* in Arabidopsis was earlier shown to lead to reduced accumulation of both Na^+^ and K^+^ in cells, with a resultant high cytosolic K^+^/Na^+^ ratio (Xu et al., 2014). The mechanism for this reduction was not explored, but the observation would suggest two things: (1) MaPIP1;1 blocks Na^+^ and/or K^+^ uptake under salt stress, and (2) MaPIP1;1 could potentially serve as a leakage point for cellular Na^+^ and K^+^, with higher selectivity for Na^+^. This however needs to be investigated.

The high-affinity K^+^ transporters (HKTs) are a family of transporters that show specificity for Na^+^ (class I), and Na^+^ and K^+^ (Class II) (Garciadeblás et al., 2003). The distinguishing feature differentiating these two classes lies in the selectivity pore-forming motif in the polypeptide, which is GGGG for class II members (found exclusively in monocots), and SGGG for class I members (found in both monocots and dicots) (Platten et al., 2006; Benito et al., 2014; Deinlein et al., 2014). It is generally believed that the G to S substitution in the first pore domain of class I HKTs restricts K^+^ permeability by steric hindrance (Cotsaftis et al., 2012), hence rendering the transporters Na^+^-selective, while the G in Class II HKTs renders them K^+^-selective, although under certain conditions they also transport Na^+^ (Mäser et al., 2002). Class I HKTs are localized mainly to the xylem/symplast boundary of roots and shoots and have been shown to mediate Na^+^ retrieval from xylem to xylem parenchyma cells. However, the rice OsHKT2;1, which is a Class II HKT based on sequence homology, is a rare example of a Na^+^-selective transporter in this class, with the SGGG instead of the GGGG motif. Unlike traditional class I HKTs, OsHKT2;1 is localized to the plasma membrane of the epidermis and mediates Na^+^ influx into the root cells, and this activity has been shown to be deleterious in rice expressing this gene (Horie et al., 2001). Among cereals, rice is the most salt-sensitive and the OsHKT2;1 is a contributing factor to this sensitivity (Horie et al., 2007). Therefore, for improved salt tolerance in rice, the down-regulation or suppression of the gene encoding this Na^+^ transporter has proven helpful. For example, the down-regulation of this gene in rice was shown to improve the tolerance of rice to salinity (Horie et al., 2007). In addition, an S to G substitution in the first pore loop of the OsHKT2;1 polypeptide resulted in a high K^+^-permeable transporter, with reduced Na^+^ permeability (Horie et al., 2007). Furthermore, *Bacillus subtilis* application to rice under salt stress resulted in a down-regulation of the *OsHKT2;1*, leading to reduced uptake of Na^+^ and a concomitant up-regulation of *SOS1* and *HKT1;5*, transcripts for Na^+^ efflux and xylem Na^+^ unloading, respectively (Niu et al., 2016). These studies indicate that alteration of OsHKT2;1 function can impact tremendously on the productivity of rice on saline soils. However, under K^+^-starved conditions and low Na^+^ concentration (<0.2 mM), OsHKT2;1 was shown to mediate nutritional uptake of Na^+^, thereby improving the growth of rice plants (Horie et al., 2007; Hauser and Horie, 2010).

**Figure 1:**
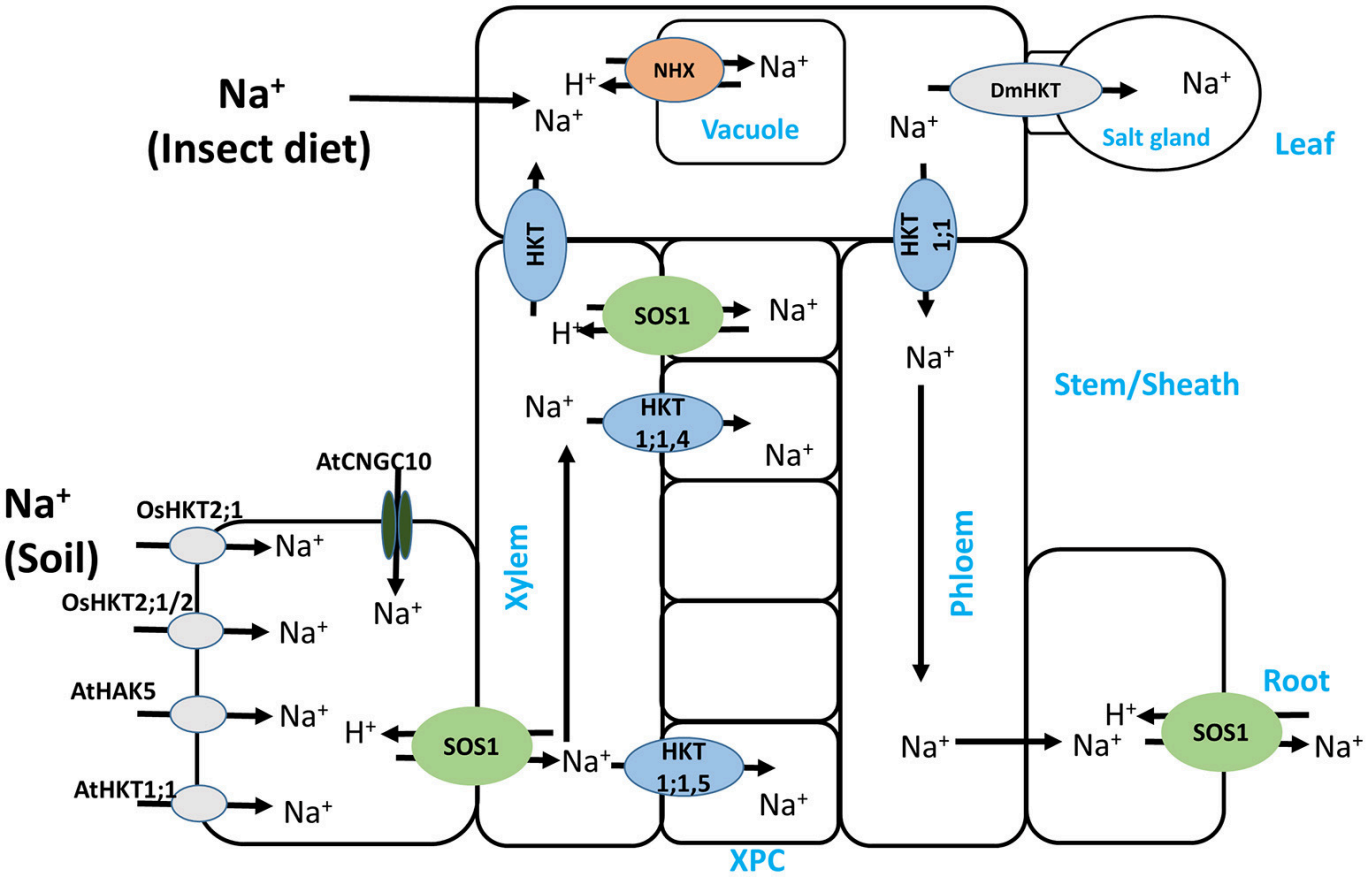
Schematic representation of Na^+^ uptake and translocation in glycophytes via Na^+^ and/or K^+^ transporters and channels. Na^+^ uptake at the leaf, as shown in the Venus flytrap plant (*Dionaea muscipula*) was not previously shown in this pathway, and its relocation to salt gland-like cells is mediated by DmHKT. In addition, OsHKT2;1/2, a hybrid of OsHKT2;1 (Na^+^-selective) and OsHKT2;2 (K^+^-selective), despite mediating K^+^ uptake, also mediates harmful Na^+^ uptake that compromises salt stress tolerance. XPC stands for xylem parenchyma cell.

HKT2;1 from other monocots including wheat (*Triticum aestivum*, TaHKT2;1), barley (*Hordeum vulgare*, HvHKT2;1) and reed (*Phragmites australis*, PhaHKT2;1), is K^+^-permeable and is important in stress tolerance. For example, Takahashi et al. (2007a) showed that the difference in tolerance between two reed species was due to an aberration in the HKT2;1 polypeptide, whereby the C-terminal region containing the fourth transmembrane domain and the M2 domain was missing, rendering the protein non-functional and hence imparting sensitivity to the species. While this study underscores the importance of HKT2;1 in alleviating salt stress, the Arabidopsis AtHKT1;1, which has been widely viewed as mediating xylem unloading of Na^+^ leading to stress tolerance, has recently been shown to be also localized to the root epidermis, where it mediates low-affinity Na^+^ uptake (Wang Q. et al., 2015). More recently, a novel HKT isoform, OsHKT2;2/1, from the salt-tolerant rice cultivar Nona Bokra, an intermediate between OsHKT2;2 (K^+^-selective transporter) and OsHKT2;1 (Na^+^-selective transporter), which was initially believed to be important for salt tolerance (Oomen et al., 2012), has now been shown to mediate damaging Na^+^ influx into the roots under salt stress (Suzuki et al., 2016a) (Figure 1). The authors suggested that although OsHKT2;1/2 mediated K^+^ uptake, the positive effect of this uptake was annulled by the large Na^+^ influx via the transporter, implying that the suppression of such a transporter gene could greatly improve stress tolerance in this rice cultivar.

**Figure 2:**
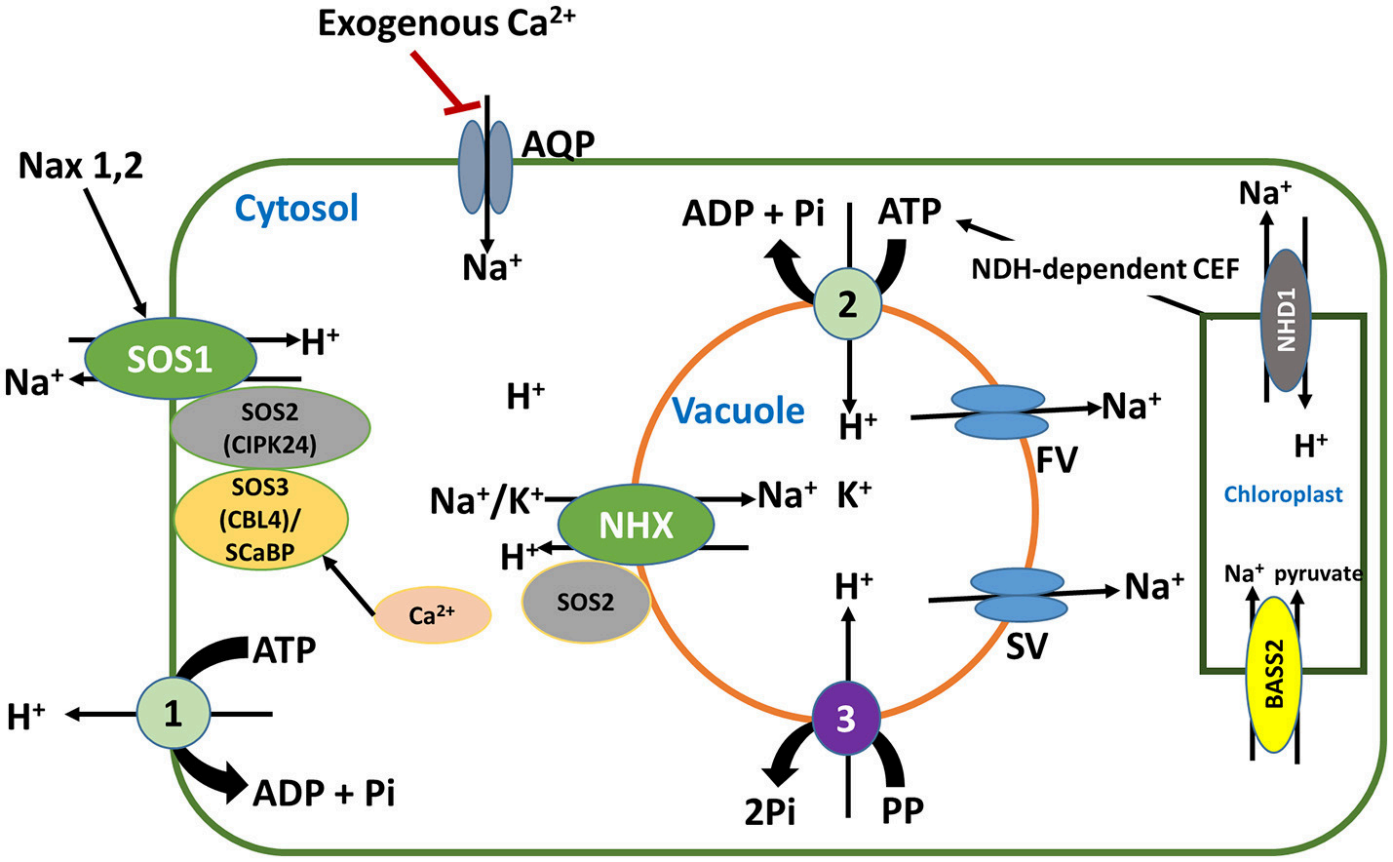
Intracellular Na^+^ homeostasis mediated by Na^+^ transporters and channels and their regulatory elements. In this figure, 1 stands for plasma membrane (PM) H^+^-ATPase, 2 stands for tonoplast (TP) H^+^-ATPase and 3 stands for TP H^+^-pyrophosphatase. Some new components in these transport mechanisms have recently been added, i.e., the regulation of stelar-localized SOS1 activity by Nax1 and Nax2 Na^+^ exclusion loci in rice. This regulation improves salt stress tolerance by enhancing the retrieval of Na^+^ from xylem back into stellar cells. Another component is the potential involvement of plant aquaporins (AQP, AtPIP2;1 in particular) in Na^+^ uptake. Equally important is the role of FV (fast vacuolar) and SV (slow vacuolar) channels that mediate vacuolar Na^+^ leakage to the cytosol, deemed a salt-sensitive trait. Worthy of note also is the role of PM and TP H^+^ pumps that generate a pmf to energize Na^+^ transport via the two Na^+^/H^+^ exchangers (SOS1 and NHXs), as well as the importance of Na^+^ efflux from chloroplasts mediated by the chloroplastic sodium hydrogen antiporter (NHD1). The NADPH dehydrogenase (NDH)-dependent cyclic electron flow (CEF) constitutes an important source of ATP required to fuel Na^+^ sequestration into vacuoles. Red line indicates inhibition, while black arrows indicate activation.

**Figure 3:**
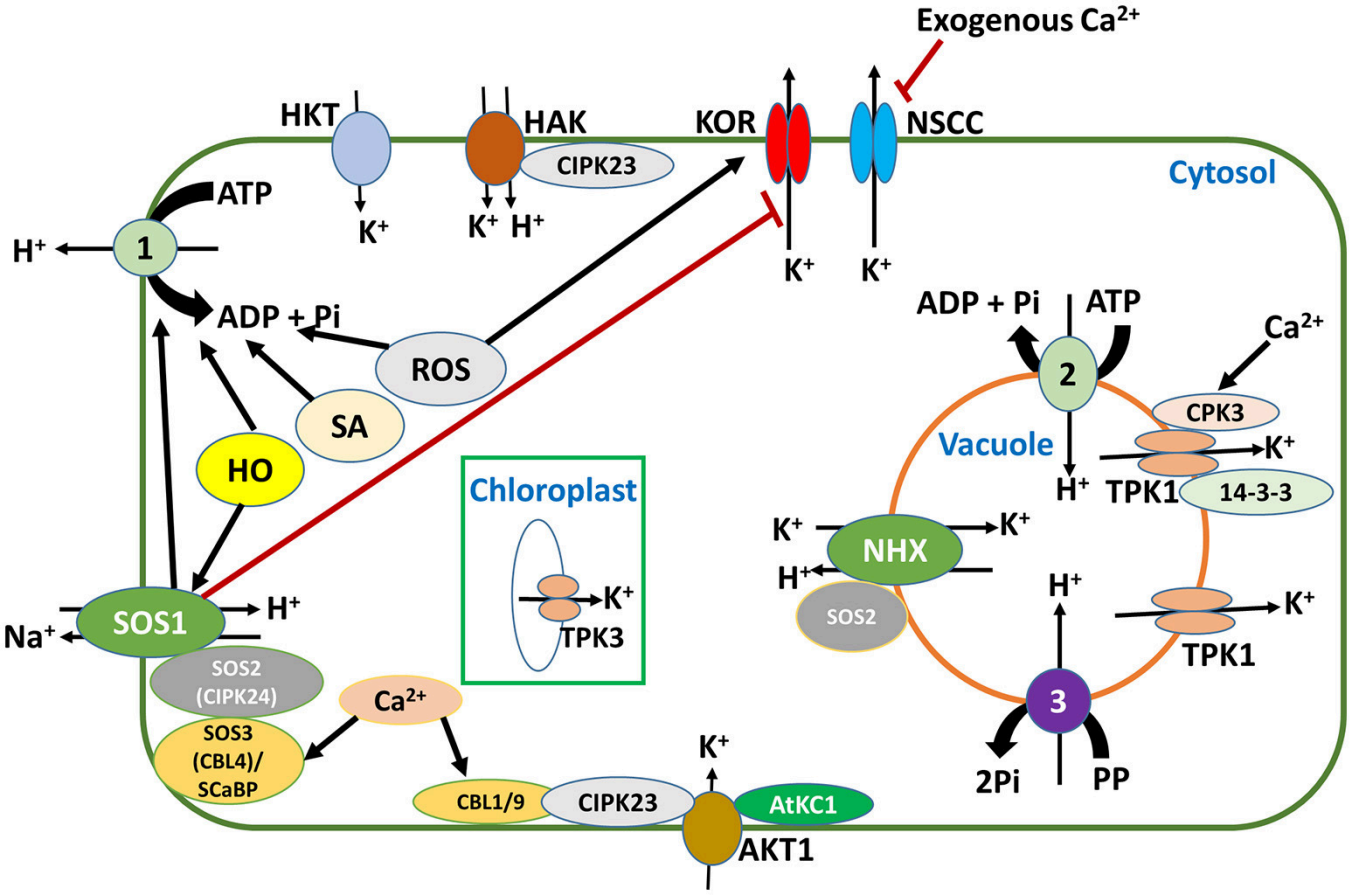
PM H^+^-ATPases are at the center of K^+^ uptake under salt stress and low-K^+^ conditions. The activity of SOS1 (salt overly sensitive 1), SA (salicylic acid), HO (haem oxygenase), and ROS (reactive oxygen species) contribute in stabilizing the membrane potential, by regulating the activity of H^+^-ATPase. This stable membrane potential is favorable for K^+^ uptake by HAK and AKT1 which are sensitive to membrane depolarization. Membrane depolarization by ROS will lead to K^+^ efflux through NSCCs (nonselective cation channels) and KORs (K^+^ outward rectifying channels), but application of exogenous Ca^2+^ and the activity of SOS1 can alleviate this condition. In the vacuole, the tonoplast two-pore K^+^ channels (TPK1) are important in replenishing lost cytosolic K^+^ from vacuolar pools, while in the chloroplast, thylakoid localized TPK3 is essential in regulating membrane potentials and protein gradients that drive ATP synthesis and the dissipation of excess energy during light photosynthetic reactions, thus enhancing plant fitness. Red lines indicate inhibition, while black arrows indicate activation.

The plasma membrane-bound K^+^ transporters and channels, *Phragmites australis* high affinity K^+^ transporters (PhaHAK2, and PhaHAK5), Arabidopsis AtHAK5 and Arabidopsis K^+^ transporter 1 (AKT1), have been reported to be involved in Na^+^ uptake (Takahashi et al., 2007b,c; Alemán et al., 2009; Nieves-Cordones et al., 2010; Mian et al., 2011b; Wang Q. et al., 2015) and so could contribute substantially to Na^+^ accumulation in plants under salt stress. For halophytes such as *Phragmites australis*, it may not be harmful, but in salt-sensitive glycophytes such as Arabidopsis, it would be deleterious. Therefore, these transporters should be considered when manipulating Na^+^ transporters to restrict Na^+^ accumulation in plants under salt stress. Noteworthy also is the regulatory network of these transporters or channels (Figures 2, 3), which can equally be targeted to control the transport activities of the transporters and channels in general.

## Resistance to radial transport and xylem loading of Na^+^

When Na^+^ is taken up at the level of the root and moves radially to the stele, it is loaded into the xylem and taken up to the shoot in the transpiration stream. Restricting the radial movement of Na^+^ across the root will greatly reduce the amount loaded into the xylem for delivery to the shoot. Using energy-dispersive X-ray microanalysis and a cryo-scanning electron microscope, Läuchli et al. (2008) determined the concentration of Na^+^ and other ions in different cell layers of wheat root. The results showed that control points for radial Na^+^ transport in the root were the cells of the cortex, pericycle and xylem parenchyma cells bordering the xylem, where there was an elevated Na^+^ concentration relative to other cell layers. In addition to this observation, the SOS1 antiporter localized to the root epidermis (especially at the root tip, where cells are undifferentiated and there is an absence of a well-formed vacuole and hence NHX), offers the first line of resistance to Na^+^ uptake, by extruding Na^+^ to the external soil environment (Shi et al., 2002). This mode of adaptation is well exemplified in the differential expression of *SOS1* between Arabidopsis (glycophyte) and *Thellugiella salsuginea* (halophyte) (Oh et al., 2009). In this study, the superior adaptation of *T. salsuginea* to salt stress is determined by enhanced expression of *SOS1* transcripts in both leaves and roots compared to Arabidopsis. Suppression of *ThSOS1* expression rendered *T. salsuginea* as susceptible as Arabidopsis to high salinity, with a high shoot Na^+^-accumulating phenotype. This phenotype resulted from lack of tight control over Na^+^ uptake that is normally ensured by WT ThSOS1. Its occurrence in the proximal (mature) part of the root has also been shown to exhibit the same function (Shabala et al., 2005). At the cortical level, OsHKT2;1 has been shown to prevent radial transport of Na^+^ at the root. Although OsHKT2;1 localized to the epidermis mediates Na^+^ influx, as mentioned earlier, it has also been shown to localize to the cortex, where it has been suggested it may intervene in compartmentalizing Na^+^ in the cortical cells, thereby limiting the amount reaching the xylem (Horie et al., 2007). A similar function has also been ascribed to AtHKT1;1 in the root, where its cortical as well as epidermal localization was found to be important for mediating Na^+^ retrieval from xylem and sequestering it in cortical cells, a process energized by the vacuolar H^+^-translocating pyrophosphatase (Craig Plett and Møller, 2010). In the stelar region of the root, HKT, for example AtHKT1;1, *Triticum monococcum* TmHKT1;5 and OsHKT1;5 localized in the xylem parenchyma cells (XPCs) bordering the xylem, retrieves Na^+^ from the xylem in to the XPCs (Møller et al., 2009; Cotsaftis et al., 2012; Munns et al., 2012).

Maintaining minimal shoot Na^+^/K^+^ ratios is an important stress tolerance trait in some halophytes and tolerant glycophytes (Munns and Tester, 2008; Katschnig et al., 2015). To achieve this, the regulation of xylem loading at the root is crucial. SOS1 localized to the stele is believed to mediate xylem loading of Na^+^ in both glycophytes and halophytes, especially under high salinity (Shi et al., 2002; Munns and Tester, 2008; Shabala, 2013). This function appears to be very important for salt-accumulating halophytes, such as *Salicornia spp*, in which the enhanced expression of *SOS1* in the root is essential to maintain a constant flow of Na^+^ via the xylem to the shoot (Yadav et al., 2012; Katschnig et al., 2015). Obviously, this situation will be lethal for salt-sensitive glycophytes. However, in some tolerant glycophytes, xylem Na^+^ loading is not necessarily harmful. For example, in *Ocimum bacilicum*, Mancarella et al. (2016) showed that leaf Na^+^ accumulation favored salt stress adaptation and preserved the photosystem's functionality under salt stress. In addition, HKTs have been shown to be implicated in xylem loading of Na^+^. Mian et al. (2011a) showed that barley plants overexpressing the *HvHKT2;1* gene resulted in enhanced xylem Na^+^ loading and consequently elevated shoot Na^+^ accumulation, which correlated positively with improved salt stress tolerance in the transgenic plants. It was however not determined in this study whether HvHKT2;1 is directly involved in xylem Na^+^ loading. However, recently it was shown in wheat that the *Nax1* and *Nax2* loci coding for HKT1;4 and HKT1;5, respectively, are involved in Na^+^ retrieval from xylem into xylem parenchyma cells in root and shoot, and are involved in the regulation of SOS1 activity in xylem loading of Na^+^ (Zhu et al., 2015). In this study, *Nax1* and *Nax2* null mutants had down-regulated *SOS1* expression with Na^+^ retrieval from xylem back to stelar cells with a resultant increase in Na^+^ accumulation in the root for osmoregulation. This finding indicates a useful link between SOS1 and HKT pathways for salt stress tolerance. It is possible, therefore, that such a link would also exist in barley, with HvHKT2;1 likely to enhance the activity of SOS1 to load Na^+^ into the xylem for translocation to the shoot for osmoregulation, in a similar way to halophytes.

The SOS pathway is a three-component system that is generally viewed as one of the most important pathways involved in Na^+^ homeostasis and salt tolerance in plants (Shi et al., 2002, 2003; Oh et al., 2009, 2010; Shang et al., 2012; Ji et al., 2013; Ma et al., 2014; Katschnig et al., 2015), with some exceptions. The first component is SOS3, a calcium-binding protein located in the plasma membrane, which upon sensing changes in cytosolic calcium signals binds the regulatory (auto-inhibitory) domain of the serine/threonine protein kinase SOS2 (second component), thereby activating and recruiting it to the plasma membrane to phosphorylate SOS1, a Na^+^/H^+^ antiporter, stimulating it to extrude Na in exchange for H^+^ (Halfter et al., 2000; Shi et al., 2002; Gong et al., 2004). This pathway occurs in both the shoot and the root, with a difference in the Ca^2+^ sensor protein. In the root the sensor is SOS3 (calcineurin B-like 4, CBL4), while in the shoot it is replaced by another ortholog of SOS3, the SOS3-like Ca^2+^ binding protein, SCaBP or CBL10. These differences were identified in mutants lacking each of the two proteins. While *scabp8* mutants showed an impaired shoot phenotype, those of *sos3* showed impaired root phenotypes (Quan et al., 2007). Furthermore, the same study demonstrated that neither SOS3 nor SCaBP8 complemented the defects of each other in the yeast system. Although in the SOS pathway SOS1 is effectively involved in Na^+^ transport, the SOS3/SOS2 or SCaBP/SOS2 complex is a determinant of its antiporter activity, implying that functional studies on SOS1 activity would require the other two components (Quintero et al., 2002) (Figure 2). However, it is now possible to circumvent this requirement, as it has been shown that deleting the C-terminal domain (auto-inhibitory and SOS2 phosphorylation site) of the *Triticum durum* TdSOS1 renders the protein more hyperactive than the native protein and functions in a way that is independent of SOS3/SOS2 phosphorylation (Feki et al., 2011, 2014). Overexpression of the truncated *TdSOS1* protein in Arabidopsis showed superior tolerance to Na^+^ and Li^+^ stress compared with WT *TdSOS1* and *AtSOS1* (Feki et al., 2014). These studies on TdSOS1 show that contrary to initial reports of the function of SOS1 as a Na^+^/H^+^ antiporter (Feki et al., 2011, 2014), it is now clear that at least in TdSOS1, the function also includes Li^+^ efflux. Furthermore, Garciadeblás et al. (2007) showed that SOS1 mediated low-affinity K^+^ transport in *Escherichia coli*, but it failed to complement K^+^ uptake deficiency in yeast, which leads to the question: is SOS1 involved in K^+^ nutrition? If this is the case, what is its role in the process? As will be seen later, SOS1 activity is very important for K^+^ uptake, especially under low-K^+^ conditions. Recently, it has been consistently shown that the difference between tolerant reed and sensitive rice is due to the difference in ability to reduce transport of Na^+^ to the shoot; only the reed plants restricted Na^+^ transport to the shoot base while the rice plants lacked this capacity (Fujimaki et al., 2015). This restriction was shown to involve Na^+^ retrieval from the xylem and recirculation to the soil through the phloem. Although SOS1 activity was not evaluated in this study, it was suggested that it might be involved in the extrusion of Na^+^ from the root. In addition, this efflux was found to be more enhanced in root tips than in bulk root, indicating that generally the site of Na^+^ extrusion mediated by SOS1 would be the meristematic non-vacuolated root tip, although other studies have found no difference in extrusion between root tips and proximal regions (Shabala et al., 2005). In barley, radiotracer analysis revealed that Na^+^ efflux mainly occurred in the root tip (three to four times that of proximal regions) of WT, while *sos1* mutants showed reduced efflux in the root tip with no alteration in efflux at other regions, indicating SOS1-mediated efflux at the root tip and mainly apoplastic release in the bulk root (Hamam et al., 2016). While this concept of Na^+^ extrusion mediated by SOS1 is widely accepted as a salt tolerance strategy, it was earlier viewed to be potentially deleterious for plants under salt stress, in the so-called “rapid transmembrane sodium cycling (RTSC)” (Malagoli et al., 2008; Britto and Kronzucker, 2015; Hamam et al., 2016). In this strategy, almost-uncontrollable Na^+^ influx into plant roots is rapidly extruded back into the soil, a process believed to be mediated by SOS1 and deemed energetically costly for the plants. This procedure is estimated to consume more ATP than is produced by respiratory processes in the root, thus creating energy deficits for other metabolic processes and consequently growth impairment (Malagoli et al., 2008; Pedersen and Palmgren, 2017). However, it has been recently suggested that a larger component of this flux would be apoplastic, and thus of minor energy cost for the root (Britto and Kronzucker, 2015; Hamam et al., 2016). However, this apoplastic fraction of the efflux has not been fully characterized. Even if this efflux were to be mainly cytosolic, it would still be an energy burden, especially for efflux mainly at the root tips, as shown by radiotracer analysis of barley and Arabidopsis roots, since the respiratory rate at this region is higher than in other parts of the root (Hamam et al., 2016).

Regulating the loading of Na^+^ into the xylem is a crucial component of salt stress tolerance. Once Na^+^ has been taken up and radially transported to the stelar region, then xylem loading becomes inevitable if there is no barrier at the xylem/symplast boundary of the root in the areas where HKT and SOS1 are localized (Shi et al., 2000, 2002; Horie et al., 2005). SOS1 under high salinity mediates Na^+^ retrieval from the xylem, but under mild stress it mediates Na^+^ xylem loading (Shi et al., 2002; Yue et al., 2012). While the notion of xylem unloading of Na^+^ by SOS1 has been contested on the basis of thermodynamic feasibility (Munns and Tester, 2008), xylem Na^+^ loading involving SOS1 is the generally accepted mechanism, and is very important for salt-accumulating halophytes (Yadav et al., 2012; Katschnig et al., 2015). HKT1 on the other hand is well known to function in the root, in regulating xylem Na^+^ concentrations by retrieving Na^+^ from the xylem into surrounding xylem parenchyma cells. For example, the Arabidopsis AtHKT1;1 (Berthomieu et al., 2003; Horie et al., 2005, 2007), rice OsHKT1;5 (Cotsaftis et al., 2012; Horie et al., 2012), wheat TmHKT1;5D (Munns et al., 2012) and huckleberry, *Solanum scabrum* SsHKT (Assaha et al., 2015), have all been shown to mediate Na^+^ exclusion by retrieving xylem Na^+^ into root xylem parenchyma cells. This process significantly reduces the amount of Na^+^ transported in the xylem to the shoot (Na^+^ exclusion).

## Restriction of Na^+^ transport to the leaf

Once Na^+^ is loaded into the xylem, it is transported via transpiration to the leaf. HKT and SOS1 found in the stem, mainly of dicots, and HKT (HKT1;4) in the sheath of monocots, participate in reducing the amount of Na^+^ reaching the leaf, by retrieving Na^+^ from the xylem into xylem parenchyma cells, thereby regulating Na^+^ delivery to the leaf blade (Horie et al., 2012). Class I HKT transporters are located at the xylem/symplast boundary and show high specificity for Na^+^, thus mediating Na^+^ unloading from xylem into xylem parenchyma cells of the shoot and root. For example, the *Arabidopsis thaliana* HKT (AtHKT1;1) (in the root and shoot) (Horie et al., 2005), the rice OsHKT1;4 (in the leaf sheath) and OsHKT1;5 (in the root) (Cotsaftis et al., 2012; Horie et al., 2012) and the wheat TmHKT1;5 (Munns et al., 2012) and TmHKT1;4 (James et al., 2011) orthologs have all been shown to be effective in reducing Na^+^ transport to the leaf blade and improving salt stress tolerance. Recently, the OsHKT1;4 has been shown to locate not only to the leaf sheath during vegetative growth, but also to the stem during the vegetative stage, where it mediates Na^+^ exclusion and improves salt stress tolerance (Suzuki et al., 2016b). In addition, differential affinity and expression of HKT can underlie differential tolerance among plants through Na^+^ exclusion from leaves. For example, Almeida et al. (2014) showed that a lower affinity and higher expression of *S. lycopersicum HKT1;2* resulted in reduced Na^+^ transport and accumulation in the leaf, compared to *S. pennellii* that showed the contrary trend. This difference was attributed to amino acid variations in the HKT1;2 polypeptide from the two species. We also recently showed that differential expression of HKT, involved in Na^+^ exclusion from the leaf, of two solanaceous species (*Solanum scabrum*-tolerant and *S. melongena*-sensitive Assaha et al., 2015), and two rice cultivars (CFX18-tolerant and Juma67-sensitive, Ueda et al., 2013), accounted for differences in tolerance. These studies indicate that differential gene expression is an important defining trait of salt stress tolerance in glycophytes. On the other hand, the tomato (*Solanum lycopersicum*) SOS1 (SlSOS1), localized to the stem, has been shown to improve salt stress tolerance in tomato plants by mediating Na^+^ retention mainly in the stem (Olías et al., 2009). The mechanism of action of SOS1 in mediating Na^+^ accumulation in the stem in this study was not explored and has remained unresolved to date. If SOS1 mediates Na^+^ extrusion out of the cell, then its activity in the stem should be in favor of xylem loading, but not unloading, which is thermodynamically not feasible (Munns and Tester, 2008). Therefore, more research is needed to elucidate this mechanism.

## Regulation of toxic Na^+^ accumulation in the leaf blade

The leaf blade is the central hub of most metabolic processes in plants and so needs to be protected from Na^+^-induced damage. Therefore, under salt stress, Na^+^ reaching the leaf needs to be rapidly redistributed in a way that will not hamper any metabolic processes. One such action was shown involving AtHKT1;1-mediated Na^+^ recirculation from the leaf to the root via the phloem (Berthomieu et al., 2003; Horie et al., 2005). Although this means of Na^+^ transport was later found to be either negligible or non-existent (Horie et al., 2007), there is increasing evidence of its occurrence, at least in the root (Tian et al., 2010; Fujimaki et al., 2015). Fujimaki et al. (2015) used the positron-emitting tracer imaging system to analyse the direction of movement of radioactive ^22^Na^+^ in the roots of reed and rice plants. They found that whereas rice plants continuously transported and accumulated Na^+^ in the shoot, reed plants' roots absorbed and moved Na^+^ only to the base of the shoot, where it was retrieved and recirculated via the phloem back to the root medium. Thus, it is possible that in some plants the same mechanism would operate in the leaf and enhance tolerance to salinity. Consistent with this notion, the OsHKT2;1 was also localized to the shoot around the vascular bundle, where it was believed to mediate Na^+^ loading in the phloem for recirculation to the root (Golldack et al., 2002; Laurie et al., 2002).

Although most of the Na^+^ delivered to the leaf is taken up at the root, there has recently been a report of Na^+^ uptake at the leaf through Na^+^-rich insect diets in the Venus flytrap (*Dionaea muscipula*) (Figure 1). The Na^+^ resulting from digestion of the prey in this plant is transported and stored in glands similar to Na^+^ sequestration in halophyte gland cells, a process mediated by the flytrap HKT-Type channel DmHKT (Böhm et al., 2016). This transporter is similar to the class I HKTs with the signature SGGG pore-forming motif described earlier and is Na^+^ selective, and hence aids in protecting the leaves from accumulation of Na^+^ to toxic levels.

Although shoot Na^+^ exclusion is widely acclaimed to be one of the most important salt tolerance mechanisms, both root and shoot Na^+^ accumulation have been shown to affect plant growth under salt stress (Álvarez-Aragón et al., 2016). This study also revealed that under salt stress in Arabidopsis, not only Na^+^ accumulation is the cause for growth impairment, but also K^+^ accumulation. As suggested in the study, Na^+^ plus K^+^ over-accumulation is potentially more deleterious than Na^+^ alone, adding yet more complexities in the salt tolerance trait.

## Potassium homeostasis

Potassium is a macronutrient and the most abundant cation in plants, constituting up to 10% of plant dry mass (Véry and Sentenac, 2003). Generally, there is no difference in K^+^ requirement, or cytosolic K^+^ concentration, between glycophytes and halophytes, but there is growing evidence that post-translational modification of some K^+^ transporter regulators can induce significant differences in K^+^ concentrations that would account for differences in tolerance between the two (Himabindu et al., 2016). Under normal growth conditions, cytosolic K^+^ concentration is maintained at a constant value of about 100 mM. The maintenance of this constant intracellular concentration is crucial because K^+^ is involved in a myriad of growth, developmental, reproductive and physiological processes, including germination, osmoregulation, stomatal regulation, nyctinastic movement of leaves, enzyme activation, loading and unloading of sugars in phloem, as a counter-ion for nitrate translocation, cytosolic pH regulation, stabilization of membrane potential and protein trafficking to protein-storage vacuoles. Since salt stress often induces perturbations in the cellular K^+^ homeostatic balance, with a consequential alteration in all these physiological processes, it has become increasingly certain that maintaining a high cytosolic K^+^/Na^+^ ratio would constitute a stress tolerance strategy (Anschütz et al., 2014; Himabindu et al., 2016). This optimal K^+^/Na^+^ ratio, especially under stress, can only be achieved if the root K^+^ uptake, xylem loading for translocation to the shoot and cellular influx are enhanced, while detrimental cytosolic K^+^ efflux is restricted at the same time.

## Mechanisms favoring root K^+^ uptake

Potassium uptake at the root-soil interface proceeds mainly via two basic mechanisms. The first mechanism is the high-affinity uptake (HAT) of K^+^ in the μM range of external K^+^ concentration, mediated by members of the KT/HAK/KUP family of K^+^ transporters such as HAK5 and KUP7. The second mechanism is the low-affinity K^+^ uptake (LAT) in the mM range of external K^+^ concentration, mediated by members of the shaker family of K^+^ channels, such as AKT1 (Gierth and Mäser, 2007; Coskun et al., 2016; Nieves-Cordones et al., 2016b) (see also Véry and Sentenac, 2003 for a detailed discussion of the classification and characteristics of K^+^ transporters and channels). However, the involvement of AKT1 in high-affinity K^+^ uptake has been reported (Gierth and Mäser, 2007). The existence of these two uptake systems (LAT and HAT) ensures a constant K^+^ supply for a resultant higher internal-to-external K^+^ concentration (Demidchik, 2014), relevant for all K^+^-dependent functions. Therefore, since NaCl often inhibits K^+^ uptake from soil, it is not surprising that increased external K^+^ concentration will help alleviate NaCl-induced defects in plants (Rodrigues et al., 2013). This raises the question: how does NaCl affect K^+^ uptake, especially under low K^+^ conditions, and how can these conditions be alleviated?

The membrane potential is largely negative in order to maintain high intracellular K^+^ concentrations (Demidchik et al., 2014). Maintaining more negative (inside) membrane potential is a key factor in salt stress tolerance (Pottosin and Dobrovinskaya, 2014). For example, barley and pea plants subjected to short term stress attain the same negative membrane potential, but under prolonged stress, barley is able to sustain a more negative potential, rendering it more tolerant than pea plants which show a continuously decreasing potential (Bose et al., 2014). The negative membrane potential is maintained by the PM H^+^-ATPase activity (Himabindu et al., 2016), which is also believed to correlate with salt stress tolerance. However, the activity of this pump is regulated by post-translational modifications (phosphorylation), and it has been suggested that this may constitute a key difference in salt stress tolerance between halophytes and glycophytes, where similar expression levels produced dissimilar activity of the pump, with higher activity in the halophyte (Himabindu et al., 2016). Thus, K^+^ uptake under low-K^+^ conditions is strongly influenced by the pump activity.

Generally, under low external K^+^ concentration and in the absence of NaCl, there is strong induction of HAK, especially group 1 members of this family, which mediate K^+^ uptake at external concentrations well below 10 μM (Alemán et al., 2009). In fact, HAK is the only K^+^ uptake transporter that is known to operate in this range of external K^+^ concentrations, and this characteristic activity for high-affinity K^+^ transport renders plants expressing the HAK transporter genes very tolerant to low-K^+^ conditions. However, under combined salt stress and low-K^+^ conditions, this expression is suppressed or the activity of the transporter drastically declines and plants become very susceptible. The activity of this transporter is inhibited because Na^+^ often depolarizes the plasma membranes to values higher than the equilibrium potential of K^+^, leading to K^+^ efflux via depolarization-activated outward rectifier K^+^ channels (Pottosin and Dobrovinskaya, 2014; Bacha et al., 2015). Another factor limiting the K^+^ uptake at this range is the activity of the PM H^+^-ATPase, which is required to create a proton motif force (pmf) across the PM, by actively pumping protons out of the cell through an ATP-dependent phosphorylation process (Falhof et al., 2016). The pmf generated by this H^+^-ATPase is then used by HAK for K^+^ uptake, since HAKs are generally K^+^/H^+^ symporters (Bose et al., 2013; Pottosin and Dobrovinskaya, 2014). Therefore, limiting membrane depolarization (e.g., by restricting Na^+^ entry or by promoting Na^+^ efflux) and enhancing H^+^-ATPase activity under salt stress may enhance K^+^ uptake via HAK and thus improve tolerance to low K^+^ under salt stress conditions. It is thanks to this observation that research on K^+^ homeostasis under low K^+^ and salinity is moving toward understanding the pathways linked to K^+^ uptake via HAKs. For example, Bacha et al. (2015) demonstrated in tomato that supplemental Ca^2+^ (5 mM) during K^+^ starvation (conditions under which LeHAK5 is induced) counteracts the Na^+^-induced PM depolarization by inhibiting NSCCs (Figure 3), which are main entry routes for Na^+^ into plant root cells as well as routes for K^+^ efflux (Guo et al., 2008). Similar results on Ca^2+^ inhibition Na^+^ uptake via NSCCs have been reported in Arabidopsis root (Shabala et al., 2006). These observations point to a crucial role for Ca^2+^ in K^+^ nutrition under salt stress via regulation of K^+^ uptake pathways.

Indeed Ca^2+^ through its relay protein, the calcineurin B-like protein (CBL), has an even greater role to play in K^+^ uptake via AKT1. For example, using a voltage clamp in oocytes and root protoplasts, Xu et al. (2006) studied the role of the CBL1/CBL9-CIPK23 complex (Figure 3) in regulating K^+^ uptake under low-K^+^ conditions. They observed that under low-K^+^ conditions, CBL1/CBL9 is activated by cytosolic Ca^2+^ to interact with CBL-interacting protein kinase 23 (CIPK23), which in turn phosphorylates AKT1, causing it to take up K^+^ (Figure 3). This observation indicates that post-translational regulation of AKT1, via phosphorylation mediated by Ca^2+^, is important for adaptation to low-K^+^ stress conditions. In agreement with this result, Cheong et al. (2007) found that the same interactions are involved not only in K^+^ uptake, but also are important in the regulation of transpiration under low-K^+^ conditions. Xue et al. (2016) also found that tobacco plants overexpressing the Arabidopsis AtCIPK23 showed increased tolerance to low K^+^, showing better growth, higher K^+^ content and a higher K^+^ uptake rate than WT plants. Although these results lend support to the idea that AKT1 is involved in high-affinity K^+^ uptake, it has not been ascertained whether this function of AKT1 can be sustained under salt stress conditions as has been shown for HAK5, the main functional transporter under low-K^+^ conditions.

Another mechanism for enhancing K^+^ uptake involves the PM Na^+^/H^+^ antiporter SOS1. In patch-clamp studies, using *sos1* mutants of Arabidopsis, Qi and Spalding (2004) showed that high cytoplasmic Na^+^ inhibited K^+^ uptake by impairing the AKT1 channel. This result indicates that in the absence of a major Na^+^ efflux system, toxic influx of Na^+^ into the cytosol will negatively affect K^+^ uptake through membrane depolarization. The role of SOS1 as shown by the above study was to activate H^+^-ATPase to pump out more protons, hence generating a pmf that is used to energize Na^+^ export. This role of SOS1 in high affinity K^+^ uptake was further supported by studies on *sos1* null mutants, which displayed reduced K^+^ uptake (Horie et al., 2012; Mansour, 2014). Similarly, Bose et al. (2013) showed that the haem oxygenase (HO), an enzyme that catalyses the production of the precursors of the antioxidants bilirubin and ferritin from haem, improves the salt tolerance of transgenic Arabidopsis by maintaining a more negative membrane potential by enhancing H^+^-ATPase activity and *SOS1* transcripts (Figure 3). HO-overexpressing mutants retained more K^+^ than loss-of-function ones and decreased Na^+^-induced membrane depolarization by extruding Na^+^ via the Na^+^/H^+^ exchange activity of SOS1. Consistently, using electrophysiological studies, Chakraborty et al. (2016) showed that the differential salinity tolerance in three Brassica species resulted from higher Na^+^ efflux via SOS1 activity, more negative membrane potential maintained by the H^+^-ATPase, and reduced sensitivity of K^+^ channels to ROS. Additional evidence for the involvement of SOS1 in K^+^ uptake was shown in Arabidopsis plants overexpressing *Physcomitrella patens* SOS1 (*PpSOS1*) grown under salt stress and low K^+^ (Fraile-Escanciano et al., 2010). The plants lacking the transporter accumulated more Na^+^ and were impaired in high-affinity K^+^ uptake compared to overexpressing lines. These results clearly lend support to the involvement of the SOS pathway in K^+^ nutrition under salt stress as well as the impact of ROS in K^+^ uptake. The SOS pathway in K^+^ nutrition would likely operate as follows: the Ca^2+^ sensor CBL, isoform CBL4 (SOS3), recruits the kinase CIPK24 (SOS2) to the plasma membrane where CIPK24 activates SOS1 via phosphorylation to extrude Na^+^ in exchange for H^+^ influx (Liu et al., 2015). This pathway aids in inhibiting membrane depolarization under salt stress; a condition favorable for K^+^ uptake. This mechanism operates in the same manner as the CBL1/9-CIPK23-AKT1 interaction for direct K^+^ uptake under low-K^+^ conditions, as mentioned earlier, further underpinning the importance of Ca^2+^ in K^+^ homeostasis.

On the other hand, ROS, particularly the hydroxyl radical (OH•), is known for inducing K^+^ efflux from the root cells of Arabidopsis by activating K^+^ outward rectifier channels such as GORK, to extrude K^+^ (Demidchik et al., 2010). However, enhanced ROS generation is not always toxic. ROS generation in the NADPH-oxidase pathway in the apoplast is important for enhancing K^+^/Na^+^ ratios under salt stress in Arabidopsis. Arabidopsis mutants lacking the NADPH-oxidase isoforms *AtrbohD1/F1* and *AtrbohD2/F2*, in which ROS production is suppressed, showed an enhanced Na^+^/K^+^ ratio. This phenotype was partially rescued by the addition of H_2_O_2_ (Ma et al., 2011). This result points to the idea that the types of ROS and their site of action determines whether they play a positive or negative regulatory role. As shown by Bose et al. (2014), the majority of K^+^ efflux from pea root is caused by ROS-induced activation of NSCC.

Growth regulators, such as salicylic acid (SA), are also implicated in K^+^ nutrition. SA is a naturally occurring growth regulator, which has been shown to alleviate both biotic and abiotic stress (reviewed by Hayat et al., 2010). Exogenous application of SA improves salinity tolerance by affecting various physiological processes including photosynthesis, antioxidant scavenging, nutrient uptake and Na^+^/K^+^ homeostasis (Gunes et al., 2007; Hayat et al., 2010; Li T. et al., 2014). In the light of its role in Na^+^ and K^+^ homeostasis, Jayakannan et al. (2013) recently demonstrated for the first time the involvement of SA in counteracting NaCl-induced membrane depolarization and in reducing K^+^ efflux via the depolarization-activated K^+^ channel GORK. Specifically, plants pre-treated with SA showed enhanced activity of the PM H^+^-ATPase under salt stress, which as stated earlier is important in reducing membrane depolarization and hence preventing K^+^ efflux and maintaining suitable conditions for K^+^ uptake, and SA-treated plants had enhanced shoot K^+^ and reduced shoot Na^+^ accumulation.

It should be noted that AtHAK5 (Alemán et al., 2009; Nieves-Cordones et al., 2010; Wang Q. et al., 2015), PhaHAK5 (Takahashi et al., 2007c) and PhaHAK2 (Takahashi et al., 2007b) are all permeable to Na^+^ (as mentioned earlier), and are induced by low K^+^ conditions, but repressed under salt stress. However, as suggested by (Alemán et al., 2009), this repression is a strategy to limit toxic Na^+^ uptake via the transporters. In contrast, the *Thellungiella halophila* HAK5 (ThHAK5) transcripts are not sensitive to salt stress and mediate K^+^ uptake under salt stress. This ThHAK5 belongs to group IV of HAKs, which is relatively less permeable to Na^+^. In addition, ThHAK5 has a higher affinity for K^+^ (Km = 1.2 μM) than AtHAK5 (Km = 12.6 μM) (Alemán et al., 2009). This differential activity between AtHAK5 and ThHAK5 is not well known, but may be due to post-translational modifications such as phosphorylation, or structural differences in the polypeptides. Single amino acid substitutions in some HAKs, e.g., AtHAK5 (Alemán et al., 2014) and HvHAK (Mangano et al., 2008) confer tolerance to NaCl stress by enhancing K^+^ uptake.

HKTs, particularly members of class II, are K^+^ selective (except OsHKT2;1) and mediate K^+^ uptake under salt stress. The fact that this class of transporters is found only in monocots (Craig Plett and Møller, 2010), implies an inherently superior capacity for monocots to tolerate salt compared with dicots. However, some of the class I members are also K^+^ selective by virtue of extra amino acid residues out of the selectivity filter residues SGGG, that alter their selectivity. These include *Thellungiella salsuginea* TsHKT1;2 (Ali et al., 2012). Interestingly, these deviant HKT homologs are found mostly in either halophytes (e.g., TsHKT1;2 from *Thellungiella salsuginea* and McHKT1 from *Mesenbryanthemum crystallinum*) or salt-tolerant glycophytes (EcHKT1;2 from *Eucalyptus camaldulensis*) (Gierth and Mäser, 2007), indicating the advantage of single-nucleotide polymorphisms in inducing stress tolerance by altering the activity or selectivity of transporters.

## Enhanced xylem K^+^ loading and translocation is important for salinity tolerance

The stelar K^+^ outward rectifying (SKOR) channel and the AtKUP7 in Arabidopsis, and OsHAK1, OsHAK5 and OsAKT1 in rice, are all involved in xylem loading of K^+^ (Véry and Sentenac, 2003; Ahmad and Maathuis, 2014; Demidchik et al., 2014; Han et al., 2016; Nieves-Cordones et al., 2016b). Under salinity stress this has the very important function of ensuring sufficient K^+^ supply to the shoot. However, under water deficit conditions, SKOR transcription is inhibited by ABA to ensure reduced K^+^ loading into the xylem to maintain adequate root turgor when soils dry out (Ahmad and Maathuis, 2014). The SKOR-mediated K^+^-efflux into the xylem is subject to alteration by ROS as the voltage sensor of the channel has been shown to have residues, (e.g., C168) that are sensitive to ROS. Substitution of another amino acid for this residue leads to loss of SKOR sensitivity to ROS (Demidchik, 2014), implying that K^+^ efflux will be arrested in this condition, which may negatively affect K^+^ homeostasis under salt stress. The involvement of ROS in K^+^ homeostasis was recently demonstrated (Ma et al., 2011). In this study, T-DNA insertion mutants, *atrbohD1*/*F1* and *atrbohD2*/*F2*, in which ROS production is completely suppressed, showed reduced K^+^ and increased Na^+^ content under salt stress, and these parameters were partially restored by application of H_2_O_2_. Although the precise mechanism of ROS-enhanced K^+^ uptake was not demonstrated, a K^+^ efflux channel that is controlled by ROS, such as SKOR would be a primary candidate route of K^+^ translocation at least. Similarly, AtrbohF was also shown to be important for regulating xylem Na^+^ loading and enhancing salt stress tolerance in Arabidopsis in a transpiration-dependent manner (Jiang et al., 2012). Here also the role of transporters was not elucidated, but it was suggested that HKTs might be involved, especially in controlling xylem sap concentrations. Although these studies clearly show the importance of ROS in Na^+^/K^+^ homeostasis under salt stress, it will be important to investigate further the mechanism of Na^+^ and/or K^+^ transport under these conditions.

K^+^ translocation to the shoot is mainly via the transpiration stream in xylem vessels. At the level of the leaf, the translocated K^+^ must be transported from the xylem vessels across the vascular bundle sheath cells to immediate or distant mesophyll cells. While the movement of K^+^ from cell to cell is mainly symplastic through plasmodesmata, its transport across the bundle sheath cells has not been clearly resolved, with many hypotheses suggesting involvement of K^+^ transporters (Wigoda et al., 2014). Most of the K^+^ translocated to the shoot is often recirculated to the root via the phloem mediated by AKT2 (Véry et al., 2014; Wigoda et al., 2014). This K^+^ recirculation through the phloem, although energy-costly, is important for the loading of sugar into the phloem in the leaf and its involvement as a counter-ion for nitrate translocation at the root (Marschner et al., 1996; Anschütz et al., 2014). From the root, the K^+^ is again loaded and translocated in the xylem to the shoot and often constitutes significant portions of the xylem K^+^ (Wigoda et al., 2014).

## Regulation of damaging cellular K^+^ efflux

Perhaps the most important salt stress adaptation toward sustaining enhanced cytosolic K^+^/Na^+^ ratios is prevention of cellular K^+^ efflux, except for guard cells where K^+^ efflux via GORK is crucial for stomatal aperture regulation (Véry et al., 2014). Under normal conditions cytosolic K^+^ concentrations are maintained at constant levels of about 100 mM, but at the onset of salt stress there is rapid decline in this concentration due to K^+^ efflux via K^+^ channels in both root and leaf cells (Shabala and Pottosin, 2014). This K^+^ efflux has been mainly attributed to Na^+^-induced membrane depolarization leading to K^+^ efflux through KORCs (Pottosin and Dobrovinskaya, 2014). In the leaf, K^+^ retention in mesophyll cells has been shown to correlate with salt stress tolerance and used as an indicator to differentiate salt-tolerant and salt-sensitive barley and wheat genotypes (Wu et al., 2013, 2015). Similarly, the ability of roots to retain K^+^ also correlates with salt tolerance in wheat and its use is proposed as a marker for breeding programs (Cuin et al., 2008). A cytosolic K^+^ concentration is also kept fixed through the movement of vacuolar pools to replenish lost cytosolic levels.

## Intracellular Na^+^/K^+^ and pH homeostasis

It is widely accepted, as mentioned earlier, that maintaining high cytosolic K^+^/Na^+^ is a prerequisite for salt stress tolerance (Maathuis and Amtmann, 1999; Anschütz et al., 2014) as this ensures optimal cellular metabolic functions. Under salt stress, the competitive inhibition of K^+^ uptake by Na^+^ often leads to the Na^+^ interfering in many K^+^-dependent processes, thereby inhibiting them. For example Na^+^ replaces K^+^ in binding sites on enzymes resulting in enzyme deactivation and consequent interruption of the metabolic processes concerned (Munns and Tester, 2008). Also, the influx of Na^+^ in cells depolarizes the membranes, leading to K^+^ efflux through depolarization-activated KOR, such as GORK (Jayakannan et al., 2013). To counter this excessive Na^+^ influx, SOS1 offers the first line of defense, as discussed earlier, by actively extruding the absorbed Na^+^ back to the extracellular spaces. In xylem parenchyma cells, this extrusion will lead to the xylem Na^+^ loading depending on external Na^+^ concentration (Shi et al., 2000; Mangano et al., 2008; Hamam et al., 2016). Vacuolar sequestration of Na^+^ is another very important strategy in the regulation of cytosolicNa^+^ accumulation. However retention of the sequestered Na^+^ has been proposed as a key stress tolerance mechanism, as Na^+^ leakage back to the cytoplasm via the fast vacuolar (FV), and slow vacuolar (SV) channels, has been associated with salt sensitivity (Isayenkov et al., 2010; Bonales-Alatorre et al., 2013a; Pottosin and Dobrovinskaya, 2014). Therefore, mechanisms that will favor the uptake and transport of K^+^ and the maintenance of high cytosolic K^+^/Na^+^ ratios should be relevant to the growth and tolerance of glycophytes under salt stress.

In the cytoplasm, excess Na^+^ accumulation will be deleterious to the cell, but owing to the tonoplast Na^+^/H^+^ antiporter NHX, the Na^+^ can be sequestered in the vacuoles and used for osmotic adjustment (Apse et al., 1999; Nieves-Cordones et al., 2016b). There are many isoforms of NHX, mainly differing in their site of localization and to a lesser extent in function. The *Arabidopsis* genome contains six isoforms (AtNHX1–6). Based on subcellular localization they are divided into two classes. Class I isoforms (AtNHX1–4) are located on the tonoplast membrane, while class II isoforms (AtNHX5 and AtNHX6, and the tomato LeNHX2) occur on endosomal membranes of the Golgi, trans-Golgi network (TGN) and pre-vacuolar compartment (PVC) (Jiang et al., 2010; Bassil et al., 2012; Huertas et al., 2013; Reguera et al., 2014). However, recently, the wheat TaNHX which is predicted to be a class I NHX has been found to be located on the endosome (Xu et al., 2013), suggesting that the classification of NHXs needs to be re-examined.

Members of the NHX antiporter family have come to be regarded as key players in intracellular Na^+^/K^+^ and pH homeostasis, growth and development, stomatal functions, protein and vesicle trafficking and tolerance to abiotic stress (Jiang et al., 2010; Huertas et al., 2013; Andrés et al., 2014; Ashnest et al., 2015; Wang L. et al., 2015; Wang et al., 2016; Wu et al., 2016). These regulatory roles result from the Na^+^(K^+^)/H^+^ exchange activity of the transporters, whose mode of action has been depicted as an “alternating access” mode (Bassil et al., 2011). In this model, NHXs bind cation/H^+^ on one side of the membrane, change conformation and transport it to the other side, where it is released and another cation or H^+^ binds and again it changes conformation and delivers the entity to the other side. The cation/H^+^ binding site of these proteins has been identified as the amiloride-binding domain motif FFIYLLPPI. The binding of amiloride (a diuretic drug) to this domain inhibits cation binding, thereby inactivating the protein (Kinsella and Aronson, 1981), since the conformational change of the protein occurs only on binding of a cation or H^+^ (Bassil et al., 2012). Although the activity of NHX clearly depends on this characteristic cation binding domain, the recent discovery that the complete NHX transcript (5′UTR-CDS-UTR3′) confers more durable salt stress tolerance than the partial transcript (5′UTR-CDS) (Amin et al., 2016) suggests the existence of other uncharacterized regulatory domains of the antiporters.

## Retention of Na^+^ sequestered in the vacuole is important for salt stress tolerance

Excess Na accumulation in the cytosol under salt stress is very noxious for cells. Of paramount importance to counter this accumulation, is the function of NHXs, which mediate Na^+^ sequestration in to vacuoles with a concomitant release of vacoular K^+^ for K^+^-specific functions in the cytosol. The release of this K operates via TPK1, and FV and SV channels (Latz et al., 2013; Pottosin and Dobrovinskaya, 2014). The SV and FV channels are very important for intracellular K^+^ homeostasis (Latz et al., 2013; Pottosin and Dobrovinskaya, 2014, but as they have low selectivity for monovalent cations including Na^+^, they constitute leakage routes for vacuolar Na^+^ (Shabala, 2013), (Figure 2). Therefore, sequestration of Na^+^ into vacuoles without preventing it from leaking to the cytolsol would not be helpful to achieve stress tolerance. Thus suppression of the activity of these channels will lead to enhanced retention of sequestered Na^+^ in the vacuole and hence enhanced salt tolerance. Indeed Bonales-Alatorre et al. (2013a) studied FV and SV channels activity in young and old leaves of quinoa (*Chenopodium quinoa* Willd.) under salt stress, and showed that closed SV and reduced FV channel activity in old leaves enhanced vacuolar Na^+^ retention, corresponding with enhanced salt tolerance. In another study, Bonales-Alatorre et al. (2013b) showed that one of the differences in salt tolerance between two quinoa genotypes is the difference in activity of the tonoplast FV and SV channels, with the tolerant genotype displaying low activity compared to high activity in the sensitive one.

## NHXs function primarily as K^+^/H^+^ and not Na^+^/H^+^ exchangers

Initially, NHXs were thought to be important for salt stress tolerance because they would catalyse the sequestration of excess cytosolic Na^+^ into vacuoles, which would be used as an osmoticum for osmotic adjustment (Apse et al., 1999). However, they were later found to also mediate vacuolar K^+^ influx and hence to be important for intracellular Na^+^/K^+^ homeostasis (Jiang et al., 2010; Huertas et al., 2013; Andrés et al., 2014), and it is now becoming increasingly clear that the main role of NHXs, especially under normal conditions or conditions of low Na^+^ concentration is as a K^+^/H^+^ antiporter, with no concurrent transport of Na^+^ (Jiang et al., 2010). This role was further supported by the finding that a halophytic AlNHX, when transferred to soybean (glycophyte), mediated less vacuolar Na^+^ sequestration (Liu et al., 2014). Thus, Na^+^ transport mediated by NHX would only occur under conditions of high Na^+^ concentration. Cell turgidity is achieved by vacuolar K^+^ pools under normal conditions. Under salt stress conditions, excessive influx of Na^+^ into the cell cytoplasm occurs, which induces membrane depolarization leading to cytosolic K^+^ efflux. To replenish the lost cytosolic K^+^, K^+^ moves from vacuoles to the cytosol, via the tonoplast two-pore K^+^ 1 (TPK1) channel (Latz et al., 2013) and this phenomenon depletes vacuolar K^+^ pools and consequently decreases cell turgor due to absence of osmotic balance (Barragán et al., 2012). Therefore, the retention of cellular K^+^ should be a salt stress tolerance determinant. Indeed, overexpression of the Arabidopsis AtNHX1 in tomato was found to enhance tolerance to salinity by mediating vacuolar K^+^ and not Na^+^ sequestration, and enhanced intracellular K^+^ retention, with a concurrent accumulation of proline and sugars in the cytosol (Leidi et al., 2010; Yaish, 2015). Similarly, transgenic alfalfa overexpressing the wheat TaNHX2 decreased K^+^ efflux by reducing plasma membrane depolarization and activation of K^+^ outwardly rectifying (KOR) channels, thereby retaining more intracellular K^+^ under salt stress conditions (Zhang et al., 2015), whereas *nhx1nhx2* double mutants of Arabidopsis lacked the ability to create vacuolar K^+^ pools resulting in compromised turgor generation for cell expansion (Barragán et al., 2012).

## NHXs alone do not account for vacuolar Na^+^ sequestration

Besides maintaining cellular turgor, tonoplast-localized NHXs are crucial for many physiological and developmental stages of plants. For example, Bassil et al. (2011) showed that *nhx1nhx2* double mutants had significant growth and reproductive-organ phenotype differences from WT, including lack of anther dehiscence, and filament elongation to extend the anthers to the level of stigmas with a resultant defect in seed set due to absence of fertilization. Interestingly, in this study, treatment of the mutants with 100 mM NaCl induced filament elongation, with development of siliques containing seeds. As suggested by the authors, this phenotype is due to the substitution of Na^+^ for the lack of K^+^ in the vacuoles. However, the mechanism underlying the entry of Na^+^ into the vacuoles of these *nhx1nhx2* plants was not evaluated, but was believed to be through transporters other than NHX1 and NHX2, which are yet to be identified (Barragán et al., 2012). In the leaf, NHX1 and NHX2 are more abundant in guard cells than in mesophyll and epidermal cells, where they mediate vacuolar K^+^ sequestration for stomatal regulation and hence transpiration rates (Barragán et al., 2012; Andrés et al., 2014). Thus, it is not surprising that tonoplast-localized NHX1 and/or NHX2 is frequently associated with improved relative water content, K^+^ concentration, K^+^/Na^+^ ratio and reduced oxidative damage (Leidi et al., 2010; Bassil et al., 2011; Patel et al., 2015; Wang et al., 2016). The mechanism of vacuolar Na^+^ sequestration was demonstrated in Arabidopsis overexpressing *AtNHX1* and shown to involve vesicle trafficking to the vacuole. NHX1 localized in the vesicular membranes mediated Na^+^ influx into the vesicles, which move, fuse and deliver their load to the vacuole (Hamaji et al., 2009). In halophytes, direct uptake of Na^+^ from the apoplast into the cytosol and from thence into the vacuole through pinocytosis (not requiring an Na^+^/H^+^ exchange activity) has been reported (Shabala and Mackay, 2011). In this mechanism, invaginations of plasma membranes and tonoplasts engulf and move apoplastic and cytosolic fluids containing large quantities of Na^+^ and Cl^−^ into the cytosol and vacuoles, respectively. This mode of vacuolar sequestration appears to be energy efficient, but has not been reported in glycophytes. Na^+^ (K^+^)/H^+^ exchange activities are energy dependent as they consume energy that would otherwise be useful for metabolic activities under salt stress (Hamam et al., 2016). However, the recently discovered correlation between enhanced ATP generation in the cyclic electron flow (CEF) and increased tissue tolerance via vacuolar Na^+^ sequestration mediated by NHX, offers useful targets for improving salt stress tolerance in glycophytes. In this study, the NDH (NADPH dehydrogenase)-dependent CEF generates extra ATPs that is used by the vacuolar H^+^-ATPase to pump H^+^ into the vacuoles (Figure 2), thereby generating an outward proton gradient that is used to sequester Na^+^ into the vacuoles. This was shown for the tolerant variety S111-9, as opposed to chloroplastic accumulation of Na^+^ in the sensitive variety Melrose of soybean (He et al., 2015). This study reveals the importance of this photosynthetic process as a source of ATP to power ATP-dependent processes, such as Na^+^ (K^+^)/H^+^ exchanges, leading to salt stress tolerance, as well as the impact of limiting photosynthesis on Na^+^ homeostasis in the leaves of plants. It also points to the importance of vacuolar H^+^-ATPases in the sequestration of Na^+^/K^+^ into the vacuole.

## NHXs regulate intracellular pH and vesicle trafficking between endosomal compartments

The NHXs function in mediating Na^+^ influx in exchange for H^+^ efflux from the vacuole or endosomes makes them excellent regulators of intracellular pH, as they serve as proton leaks to counter aberrant acidification of the intracellular compartments (Bassil and Blumwald, 2014; Reguera et al., 2014, 2015). A clear example of tonoplast NHX involvement in pH regulation was shown in the Japanese morning glory, *Ipomoea nil* or *Ipomoea tricolor*, where a change in petal color from purple-red to blue is accompanied by a sudden rise in pH from 6.6 to 7.7, with a concomitant increase in *InNHX1* transcripts 12 h before flower opening. This alkalization of the vacuole was shown to be due to vacuolar accumulation of K^+^, and not Na^+^, mediated by ItNHX1, while V-PPase and V-ATPase functioned to prevent over-alkalization (Ohnishi et al., 2005; Yoshida et al., 2005, 2009).

The endosomal NHXs, NHX5 and NHX6 are co-localized to the Golgi, trans-Golgi and PVCs. These antiporters have received much attention recently as they are indispensable for various intracellular processes including pH regulation, cell expansion, osmoregulation, protein-storage vacuole (PSV) biogenesis and protein trafficking to the PSV (Li et al., 2011; Reguera et al., 2014, 2015). Single *nhx5* or *nhx6* mutants have phenotypes that are not significantly different from WT plants, but *nhx5nhx6* double mutants show clearly distinct phenotypes from the WT plants. This demonstrates that they have the same function and so complement each other in the absence of either one of them. NHX5 and NHX6 regulate endosomal pH by preventing acidification through leakage of H^+^ out of the compartments (Qiu, 2016a,b), and regulate protein transport by controlling three distinct stages: the binding of vacuolar sorting receptor (VSR), recycling of VSRs and the subcellular localization of the SNARE complex (Ashnest et al., 2015; Reguera et al., 2015; Qiu, 2016a,b; Wu et al., 2016), and they participate in protein storage vacuole formation as well as in retromer-dependent vacuolar trafficking through their cytosolic C-terminal. Deletion of this C-terminal in NHX6 failed to rescue the *nhx5nhx6* phenotype (Ashnest et al., 2015). The SNARE complex directs the fusion of PVCs to the vacuole, thereby mediating protein trafficking (Wu et al., 2016). Wu et al. (2016) showed that the three amino acid residues, D164, E188 and D193 in AtNHX5 and D165, E189 and D194 in AtNHX6 are essential for transporting the storage proteins. These same amino acid residues in NHX5 and NHX6 were earlier shown to be also essential for K^+^ homeostasis and growth under salt stress in Arabidopsis (Wang Q. et al., 2015). In addition, AtNHX5 and AtNHX6 control the trafficking of seed storage proteins to the protein storage vacuoles by regulating the subcellular localization of the SNARE complex in Arabidopsis (Wu et al., 2016). The *nhx5nhx6* mutation results in the mis-sorting of protein precursors to the apoplast (Bassil et al., 2011). The binding of cargo protein to vacuolar sorting receptors (VSRs) (Amin et al., 2016) occurs on the NPIR motif at the N-terminus of the cargo protein and this cargo-VSR interaction is pH- and Ca^2+^-dependent. Hence *nhx5nhx6* mutants show defects in proper protein processing and sorting to storage vacuoles (Reguera et al., 2015).

## Exclusion of Na^+^ from chloroplast is important for salt stress tolerance

As cytosolic Na^+^ is noxious to cells, so too is chloroplastic Na^+^ accumulation. Na^+^ is important in C_4_ plants because it is required for the transport of pyruvate into the chloroplast for the synthesis of PEP for photosynthetic CO_2_ fixation. In these plants, the co-transport of the Na^+^ and pyruvate is mediated by the Na^+^/pyruvate symporter BASS2 (bile acid/sodium symporter family protein 2), while the Na^+^ is exported by a sodium hydrogen antiporter (NHD1, Figure 2) (Furumoto et al., 2011). In C_3_ plants where there is no such role for Na^+^, accumulation of Na^+^ in the chloroplast may be deleterious. Indeed, using knockout lines of NHD1 in Arabidopsis, (Müller et al., 2014) showed that the inability to export Na^+^ out of the chloroplast can impair the salt tolerance, photosynthesis and growth of the plants. It can be inferred from the NHD1 function in this study that proton pumps should also be crucial for Na^+^ homeostasis in the chloroplast. Although not demonstrated yet, the CEF, which generates only ATP (Yamori et al., 2016), may be involved in providing energy to enhance the pump activity required for Na^+^ efflux from the chloroplast in a similar manner to vacuolar Na^+^ sequestration (He et al., 2015).

Another important regulator of intracellular K^+^/Na^+^ ratio is the TPK channel. Tonoplast-localized TPK1 in Arabidopsis is known to be activated upon CDPK phosphorylation by interaction with 14-3-3 proteins (Figure 3) and mediates cytosolic K^+^ influx from vacuolar pools during salt stress-induced cytosolic K^+^ efflux (Latz et al., 2013). Another isoform of this channel, TPK3, localized to the thylakoid membranes of the chloroplast was recently shown to not only mediate K^+^ homeostasis, but also to contribute to plant fitness by balancing the electrochemical proton gradient across the thylakoid, which is required for ATP synthesis and dissipation of excess light energy during the light phase of photosynthesis (Carraretto et al., 2016).

## Conclusion

This review has shown that Na^+^ and K^+^ transporters are very important for salt stress tolerance. However, the susceptibility of glycophytes to salt stress when compared to tolerant species or halophytes may lie in the structural nature of some of these transporters. As shown by point or site-directed mutations, changes in specific amino acids in the polypeptides can significantly alter the activity or selectivity of the transporters, and improve tolerance to salt stress. Also, post-translational modifications such as phosphorylation significantly alter the activity of some of the transporters such as SOS1 or NHXs, or regulatory components of the transporters, such as post-translational phosphorylation of H^+^-ATPases. These observations are important in understanding partly why halophytes are more salt-tolerant than glycophytes and thus could constitute useful targets for engineering salt stress tolerance. In addition, novel regulatory pathways have been uncovered which, although augmenting the complexity of salinity, could also serve as important targets for improving salt stress tolerance.

## Author contributions

All authors listed, have made substantial, direct and intellectual contribution to the work, and approved it for publication.

### Conflict of interest statement

The authors declare that the research was conducted in the absence of any commercial or financial relationships that could be construed as a potential conflict of interest.

## Abbreviations

ABA: abscisic acid
AKT1: Arabidopsis K^+^ transporter
BASS: bile acid/sodium symporter
CBL: calcineurin B-Like
CDPK: Ca^2+^ dependent protein kinase
CEF: cyclic electron flow
CIPK: CBL-interacting protein kinase
CNGC: cyclic nucleotide-gated channel
FV: Fast vacuolar channel
GORK: guard cell outwardly rectifying K^+^ channel
GRL: glutamate receptor-like
HAK: high affinity potassium transporter
HAT: high affinity transport
HKT: high affinity K^+^ transporter
HO: haem oxygenase
KORC: potassium outward rectifying channel
KT: potassium transporter
KUP: potassium uptake transporter
LAT: low affinity transport
NADPH: Nicotinamide adenine dinucleotide phosphate
NDH: NADPH dehydrogenase
NHD: Na^+^/H^+^ antiporter
NHX: Na^+^/H^+^ exchanger
NSCC: nonselective cation channel
PEP: phosphoenolpyruvate
PIP: plasma membrane intrinsic protein
PM: plasma membrane
pmf: proton motif force
PMP: plasma membrane protein
PSV: protein storage vacuoles
PVC: pre-vacuolar compartment
ROS: reactive oxygen species
RTSC: rapid transmembrane sodium cycling
SA: salicylic acid
SCaBP: SOS3-like Ca^2+^ binding protein
SKOR: stelar potassium outward rectifying channel
SNARE: N-ethylmaleimide-sensitive factor adaptor protein receptor
SOS: salt overly sensitive
SV: slow vacuolar channel
TGN: trans-Golgi network
TP: tonoplast
TPK: two-pore (or tandem-pore) K^+^ channel
VSR: vacuolar sorting receptor
XPC: xylem parenchyma cell.
